# Fused Filament Fabrication of Small Ceramic Components

**DOI:** 10.3390/ma11081463

**Published:** 2018-08-17

**Authors:** Dorit Nötzel, Ralf Eickhoff, Thomas Hanemann

**Affiliations:** 1 Karlsruhe Institute of Technology, Institute for Applied Materials, 736344 Eggenstein-Leopoldshafen, Germany; ralf.eickhoff@web.de (R.E.); thomas.hanemann@kit.edu (T.H.); 2 Laboratory for Materials Processing, University of Freiburg, 79110 Freiburg, Germany

**Keywords:** 3D printing, FFF, FDM, polymer-ceramic composite, ceramic printing, rapid prototyping

## Abstract

With respect to rapid prototyping of ceramic components, there are known only a few processes (stereo lithography, binder jetting). In this work, a new process chain is described in detail, showing that ceramics can be printed in a very cost-efficient way. We developed a ceramic–polymer composite as filament material that can be printed on a low-cost fused filament fabrication (FFF) desktop printer, even with very small nozzle sizes enabling very small geometric feature sizes. The thermal post-processing, with debinding and sintering, is very close to the ceramic injection molding (CIM) process chain.

## 1. Introduction

Powder injection molding (PIM) is a well-known technique to produce even small objects from metal (MIM) or ceramic (CIM) materials [1,2,3]. This process is very cost-efficient, especially for large quantities [2]. For small lot sizes or customized parts, like prototypes, this technique is not affordable, due to the expensive tools and longer production times [4]. One approach to get small quantities is to produce tools from plastic materials with standard three-dimensional (3D) printing methods, called rapid tooling [4,5].

Another approach is to produce prototypes without tools using additive manufacturing [6]. However, most of these methods are only for plastic [7,8,9].

There are already 3D-printing methods of ceramic materials known. Tay et al., Travitzky et al., and Gonzalez-Cutierrez et al. summarize common processes, including stereo lithography, selective laser sintering, laminated object manufacturing, three-dimensional printing, direct ceramic ink-jet printing, and fused filament fabrication [10,11,12].

Fused filament fabrication (FFF), also known as fused deposition modelling (FDM), usually uses thermoplastics. In the past, however, there were many attempts to improve or modify the thermoplastic material properties by adding small amounts of diverse powder or fiber materials [13,14,15,16,17,18]. Ceramic fillers were already being used as well, either to enhance mechanical or thermal properties of the thermoplastics [19,20], or for biological applications [21].

By filling thermoplastics with ceramic powder in very high solid contents (>45 vol %), it is feasible to print objects that can be debinded and sintered to dense ceramic parts. In the literature [22,23,24] a procedure is described whereby ceramic powders are dispersed in a self-developed binder system, printed with a commercial Stratasys printer, debinded in flowing nitrogen, and finally sintered. Since these publications almost 20 years ago, there can hardly be found anything related to printed and sintered dense ceramics in literature. Quite recently, feedstocks for CIM were introduced also for 3D printing [25,26], and a review was published by Gonzalez-Gutierrez et al. [12]. With respect to these publications, we developed a process chain to use common inexpensive FFF printers to print small ceramic components.

Concerning the fine geometric details of the print, filament materials require special properties, which are, to a certain extent, comparable to feedstock properties for micro powder injection molding (µPIM) [2,27]:

- molten feedstock viscosity should be low for low-force nozzle extrusion,
- high strength of feedstock at room temperature for good filament and printed object stability,
- complete debinding and sintering of the parts without deformation,
- solid loads between 45 and 60 vol % for high densities and warpage-free shape after sintering,
- particle sizes of a maximum of 10% of the aspired structural details,
- complete binder wetting of the particles for best deagglomeration.

In order to print the new feedstock materials, the binder system melting temperature should necessarily fit the heater temperature of the nozzle [28]. Furthermore, the slicer software for material development has to be open, which provides an opportunity for independent adapting of printing parameters like temperature, speed, layer thickness, nozzle size, etc., by the user.

## 2. Materials and Sample Preparation

### 2.1. Materials

**Figure 1:**
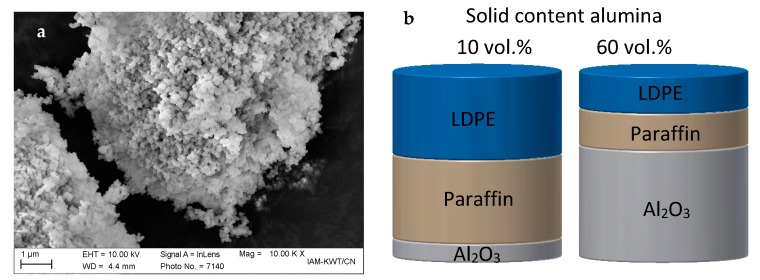
(**a**) SEM image of Al_2_O_3_; (**b**) scheme of the feedstock composition exemplarily for 10 and 60 vol % alumina.

A sub-micron-sized alumina (Al_2_O_3_, TM-DAR, Tamai Chemicals, Tokyo, Japan) was chosen as the ceramic powder. The specific surface area measured following the BET-method was 12 m^2^/g (Gemini VII 2390, Micromeritics Instrument Corp., Norcross, GA, USA), and the particle size d_50_ was 0.1 µm (LA-950, Horiba Ltd., Kyoto, Japan). An SEM image of the alumina filler is shown in Figure 1a. 

Even if the final components consist of pure ceramic, the thermoplastic binder system is a temporary vehicle to enable shaping and stabilization of the ceramic particles [27]. In our study, the filament materials consist of 10 up to 60 vol % of ceramic filler (32–86 wt %). The rest of the volume splits into paraffin, Low Density Polyethylene (LDPE, melt flow rate 1.5 g/10 min at 190 °C, melting temperature 108 °C), and stearic acid as surfactant and release agents in a constant ratio. A schematic diagram showing the composition is given for both the 10 and 60 vol % materials is shown in Figure 1b.

### 2.2. Compounding

**Figure 2:**
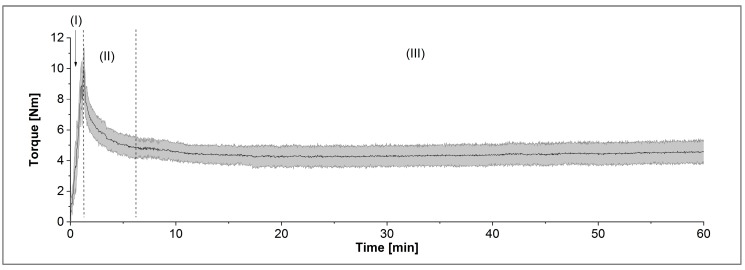
Principal mixing diagram with (I) filling process, (II) mixing state, and (III) equilibrium.

The composites of thermoplastic polymers and ceramic powders were prepared in a Brabender measuring mixer-kneader (W50 EHT, Brabender Instruments, South Hackensack, NJ, USA) as described in [29]. The temperature was set to 125 °C, and the mixing torque was recorded during the compounding time of 60 min. The curves can be divided principally into three sections: (I) the filling process, (II) the mixing and wetting state, and (III) the equilibrium state, as shown in Figure 2. The grey area shows the range of absolute values during a measuring period of two seconds. The latter delivers the final torque value, which can be treated as a first hint if the composite can be printed or not.

The shear rate-dependent melt viscosity of all composites was measured in a high-pressure capillary rheometer (RG 25, Goettfert GmbH, Buchen, Germany) with a capillary diameter of 1 mm and a length of 30 mm, at a temperature of 160 °C.

### 2.3. Preparing Filaments

**Figure 3:**
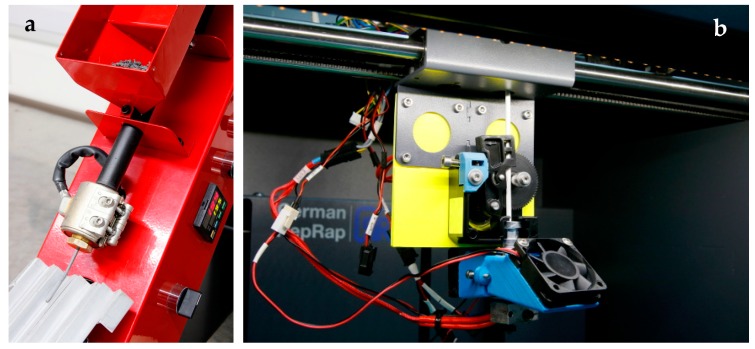
(**a**) Filament extruder, (**b**) modified fused filament fabrication (FFF) printer head.

Filaments were prepared with a one-screw extruder (Noztek pro, Noztek, Shoreham, UK), as shown in Figure 3a, at temperatures of 100–130 °C. The nozzle diameter was selected to reach filament diameters of approximately 3 mm. Because the polymer–ceramic composites are too stiff and brittle to be winded, the filaments were cut every 50 cm.

With these filament dimensions, dense green bodies of sizes up to 3.5 cm^3^ can be realized. In order to get larger samples, a filament changing unit is conceivable.

### 2.4. Printing via Fused Filament Fabrication

All samples were printed in a slightly modified desktop FFF printer (X350 pro, German RepRap, Bayern, Germany). The modifications concern the filament size of 3 mm instead of 1.75 mm, as well as a Titan extruder with a gear ratio of 3:1. In Figure 3b, the modified printing head is shown. The slicing of the parts for printing was done using Simplify3D and Cura.

### 2.5. Post-Processing

**Figure 4:**
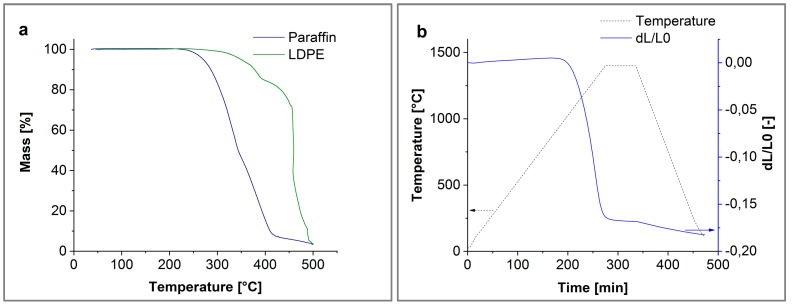
(**a**) Thermographimetric analysis (TGA) of paraffin and LDPE, and (**b**) TGA of dilatometer Al_2_O_3_.

After printing, the polymer binder had to be removed, which enables successful sintering at higher temperatures. First of all, the paraffin was dissolved in n-Hexane. This initial solvent treatment opens pores, while the LDPE remains and retains the shape of the printed green part [27]. In the subsequent thermal debinding step, the remaining LDPE was removed. In Figure 4a, the thermographimetric analysis (TGA) of the used paraffin and LDPE is shown. With respect to a controlled mass loss, suitable heating rates can be determined. Even though at 500 °C some polymer traces are still present, the debinding process was stopped to guarantee mechanical stability to move the objects into the sintering oven. In Figure 4b, the dilatometer measurement of the alumina is shown. Therefore, TM-DAR already sinters at 1400 °C. The debinded samples were sintered at 1400 °C for 1 h, and the test discs at 1400 °C for 6 h, as introduced in [30].

## 3. Results and Discussion

### 3.1. Compounding and Rheological Investigation

**Figure 5:**
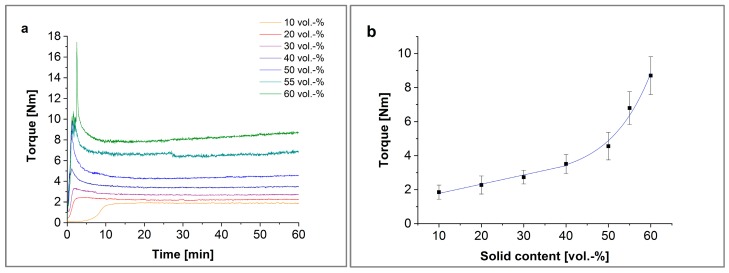

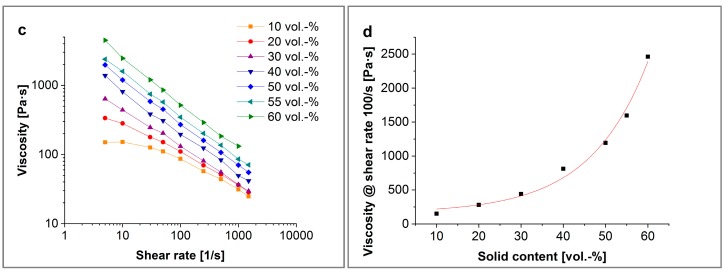
(**a**) Kneading diagram at 125 °C, (**b**) ending torque at 125 °C, and (**c**) viscosity at 160 °C, and (**d**) viscosity at 100 s^−1^ at 160 °C.

The powder agglomerates are destroyed during compounding, and the primary particles are dispensed homogenously in the polymer binder, observable by means of a smooth curvature during kneading (Figure 5a). The torque that the kneader needs to maintain the mixing speed depends on the solid content and the homogeneity of the materials. In Figure 5a,b are shown the torque profiles and the ending torque at 60 min mixing time.

The solid content of 10 and 20 vol % material has no mixing peaks. The solid contents are too low for a direct interaction of the ceramic particles with one other. Until 30 vol % the torque of the equilibrium state increases linearly, from 40 vol % the torque of the mixing state increases exponentially (Figure 5b). With increasing solid content, further ceramic particles grind while deagglomerating and wetting with the polymer binder.

In Figure 5c, the rheological measurement over a broad shear rate range is shown. Like in the torque profile, the measurements of 10 and 20 vol % are different from the higher loaded samples. At very low shear rates, the zero viscosity (first Newtonian plateau) of the polymer binder system can be observed. However, all samples show pronounced shear thinning behavior, as expected in powder-filled polymer systems. Figure 5d shows the viscosity at a shear rate of 100 s^−1^, which is comparable to the printing shear rate in FFF. The viscosity increases exponentially more than one order of magnitude from 10 to 60 vol %.

### 3.2. Filament Preparation

**Figure 6:**
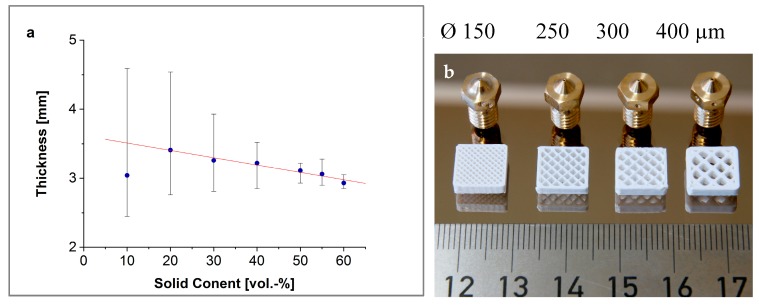
(**a**) Filament diameter as function of solid content, and (**b**) open test structures printed with different nozzle sizes.

For exact printing results, the filament diameters must be within a very small tolerance range, as already observed in Section 2.3. The thickness was measured every 5 cm (10 points per filament) along 6–10 different filaments. The filament diameters decreased linearly with increasing solid content (Figure 6a). This can be explained due to the usual die swell of viscoelastic polymers. The higher the solid content of the material, the lower the polymer volume that swells at the end of the nozzle. With increasing solid content, the absolute errors (error bars in Figure 6a) and the standard deviations minimizes as well (Table 1).

Due to the significant inhomogeneities of the filament diameters up to a solid content of 40 vol %, only materials ≥50 vol % were selected for printing. Filaments with diameters of more than 3 mm were extruded again with a smaller nozzle size.

### 3.3. Printing

In order to print small components with geometric features around or smaller than 100 µm, fine nozzle diameters are crucial. Examining the printability of the compounds, different nozzle sizes of 150, 250, 300, and 400 µm were tested. In Figure 6b, an open test structure of 10 × 10 × 2.5 mm³ is shown. All structures are printed at nozzle temperatures of 150–170 °C, a layer thickness of 100–200 µm, and an infill of 25%. Due to the different nozzle sizes, the bar width in the structure in combination with the layer thickness fills more or less the object volume. The smaller the nozzle, the more bars were printed.

**Figure 7:**
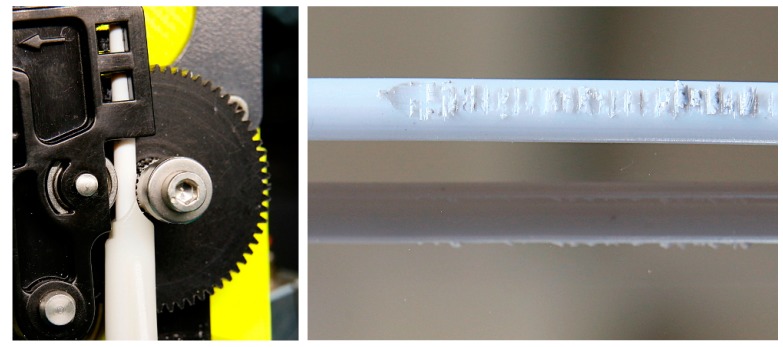
Grinding of a filament containing 60 vol % alumina.

The filament with a solid content of 50 vol % alumina shows a good printing behavior. The material with 55 vol % solid content can be printed with a nozzle size of 400 µm. The highest filled material, with 60 vol %, can be extruded manually only, but the viscosity is too high to be extruded by the printer. Because the extruder gear is unable to move the filament, grinding at the surface occurs, as can be observed in Figure 7. However, nozzle clogging with increasing solid content as described of Kariz et al. in [17] could not be observed.

Concerning finely detailed prints, the nozzle size has to be as small as possible. Due to the smallest nozzle currently available for X350 printers of a diameter of 150 µm, only the composite filled with 50 vol % alumina can be used. The best printing results could be achieved with a printing temperature of 160 °C, a speed of 5 mm/s, and a layer thickness of 0.1 mm.

The material with a solid content of 50 vol % was used to print dense disks with a diameter of 10 mm and a height of 2 mm. Due to the round or oval profile of the extruded filament, it is a challenge to fill objects to 100% without pores, as already described by Agarwala and Qui [23,31]. 

### 3.4. Post-Processed Samples

**Figure 8:**
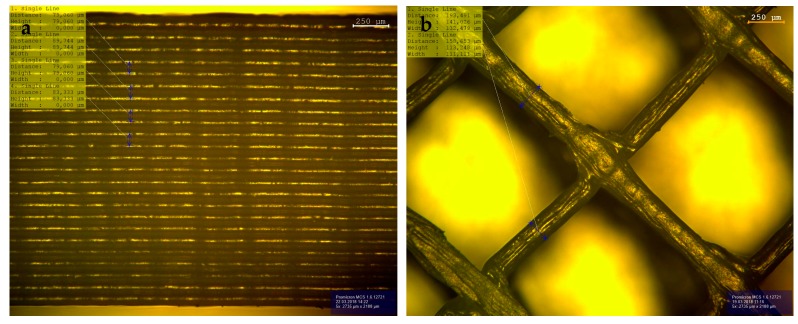
(**a**) Side view and (**b**) top view of the open test structure.

**Figure 9:**
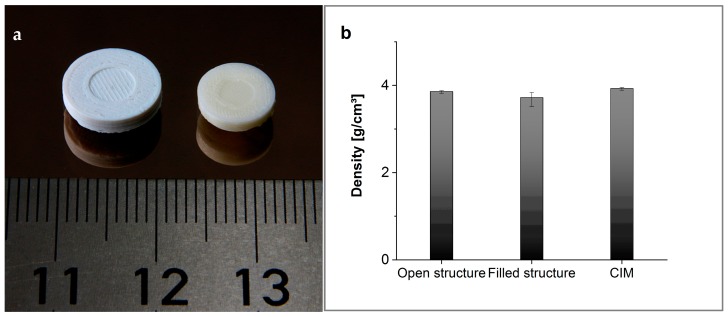
(**a**) Test disk and (**b**) density of different samples.

After printing, all samples were debinded and sintered as described above. In Figure 8, light microscopic pictures of a sintered open structure of 50 vol % alumina are shown. The sample was printed with a nozzle size of 150 µm and a layer thickness of 100 µm. After sintering, the layer thicknesses achieve 80 to 90 µm, and the bar reaches a width of 160 µm. In Figure 9b, the densities of all sintered samples are shown. The density of the sintered open-structured samples reaches up to 98.4% of the theoretical density.

Test discs with 100% infill were debinded and sintered together with disks prepared by ceramic injection molding (CIM), applying the same highly-filled composite (denominated “feedstock” in CIM) to compare the resulting sinter densities. A green and a sintered printed test disc are shown in Figure 9a. While the CIM samples reach densities up to 99.7% of the theoretical density, the printed sample has values up to 97.3% of the theoretical density.

**Figure 10:**
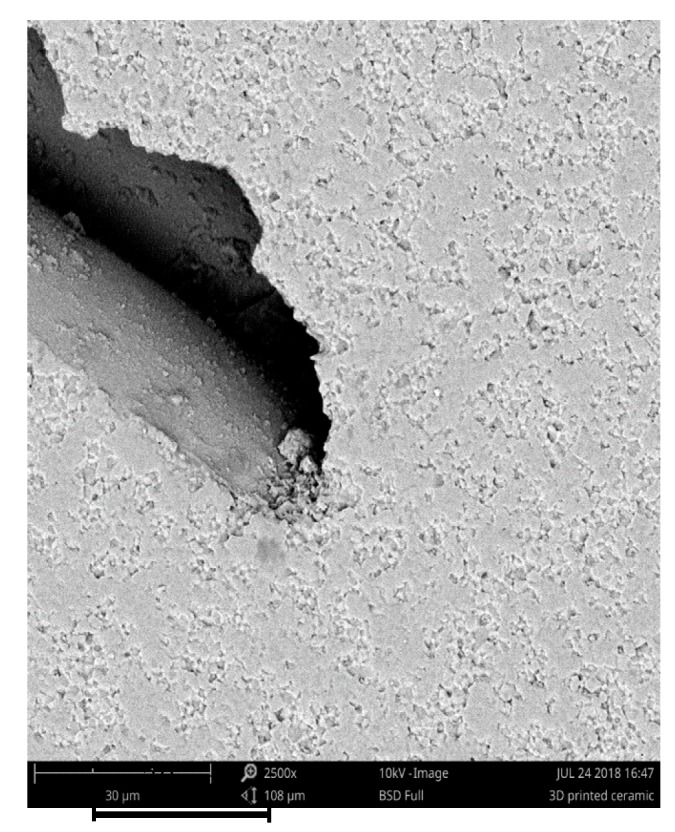
SEM image of a generated cavity.

As suspected, the CIM samples showed the highest densities, because this process delivers a massive specimen without any pores, in contrast to the layer-by-layer printed specimen. The filled printed samples showed poorer densities and higher deviations, because pores and cavities are generated while printing. Figure 10 shows an example of such a generated cavity. Two walls of extruded filaments can be seen. Due to the toolpaths generated by the slicers, the pore is not closed by the next layer. This verifies the higher densities and lower deviations of the printed open structures. Because of the thin walls, there are not many opportunities to generate pores.

## 4. Conclusions

The FFF 3D printing method is the most common 3D printing method, due to its inexpensive printers and the wide range of used materials. We developed a very highly-filled filament material and introduced a complete process chain to print ceramic components via FFF.

The mixing torque and the viscosity of the materials increased with increasing solid content. Filament diameters and standard deviations decreased with increasing solid content.

Materials with a solid content of 50 vol % are very printable with nozzle sizes down to 150 µm. Layer thicknesses of 80 µm and bar widths of 160 µm could be realized with open demonstrator structures.

Open structures have sintering densities of 98.4% of theoretical density; test discs have only 97.3%, while ceramic injection molded samples reach 99.7%. Probably during printing process, pores and cavities are introduced, due to not completely filling tool path generation by the slicing programs. As described by Qui and Langrana [31], voids could be eliminated by intelligent path management.

Because our introduced process chain is tool-free, it is a strong and promising way to produce prototypes or small amounts of ceramic objects. As an additional feature, the newly developed composite is suitable for usage in FFF/FDM, as well as in ceramic injection molding.

As alumina can be replaced with other ceramics, a very wide product range occurs, where only the printing and sintering parameters have to be adapted.

According to these results, it is feasible to generate prototypes and design studies in a simple and cost-efficient way.

## Figures and Tables

**Table 1:** Filament diameters.

Solid Content [vol %]	Average Diameter [mm]	Standard Deviation [mm]
10	3.04	0.57
20	3.41	0.42
30	3.26	0.26
40	3.22	0.13
50	3.11	0.07
55	3.06	0.07
60	2.93	0.03
